# Author Correction: Intestinal microbiome adjusts the innate immune setpoint during colonization through negative regulation of MyD88

**DOI:** 10.1038/s41467-019-08456-y

**Published:** 2019-01-28

**Authors:** Bjørn E. V. Koch, Shuxin Yang, Gerda Lamers, Jens Stougaard, Herman P. Spaink

**Affiliations:** 1 Institute of Biology, Leiden University, 2333 BE Leiden, The Netherlands; 2 Department of Molecular Biology and Genetics, Aarhus University, 8000 Aarhus C, Denmark; 3 Present Address: Center for Synthetic Biology Engineering Research, Shenzhen Institutes of Advanced Technology, Chinese Academy of Sciences, 518055 Shenzhen, China

Correction to: *Nature Communications*; 10.1038/s41467-018-06658-4; published online 05 October 2018.

The original version of this Article contained an error in the spelling of the author Shuxin Yang, which was incorrectly given as Shuxing Yang. This has now been corrected in both the PDF and HTML versions of the Article.

