# Supplementary material for: Intestinal microbiome adjusts the innate immune setpoint during colonization through negative regulation of MyD88
Source: Nat Commun. 2018 Oct 5;9:4099. doi: 10.1038/s41467-018-06658-4 (PMC6173721; doi:10.1038/s41467-018-06658-4)
Supplement: Supplementary file 1 — Supplementary Information [file 41467_2018_6658_MOESM1_ESM.pdf]

Supplementary information

Intestinal microbiome adjusts the innate immune setpoint during colonization through negative regulation of MyD88

Koch et al.

Supplementary figures 1-7

Supplementary tables 1-8

Supplemental material

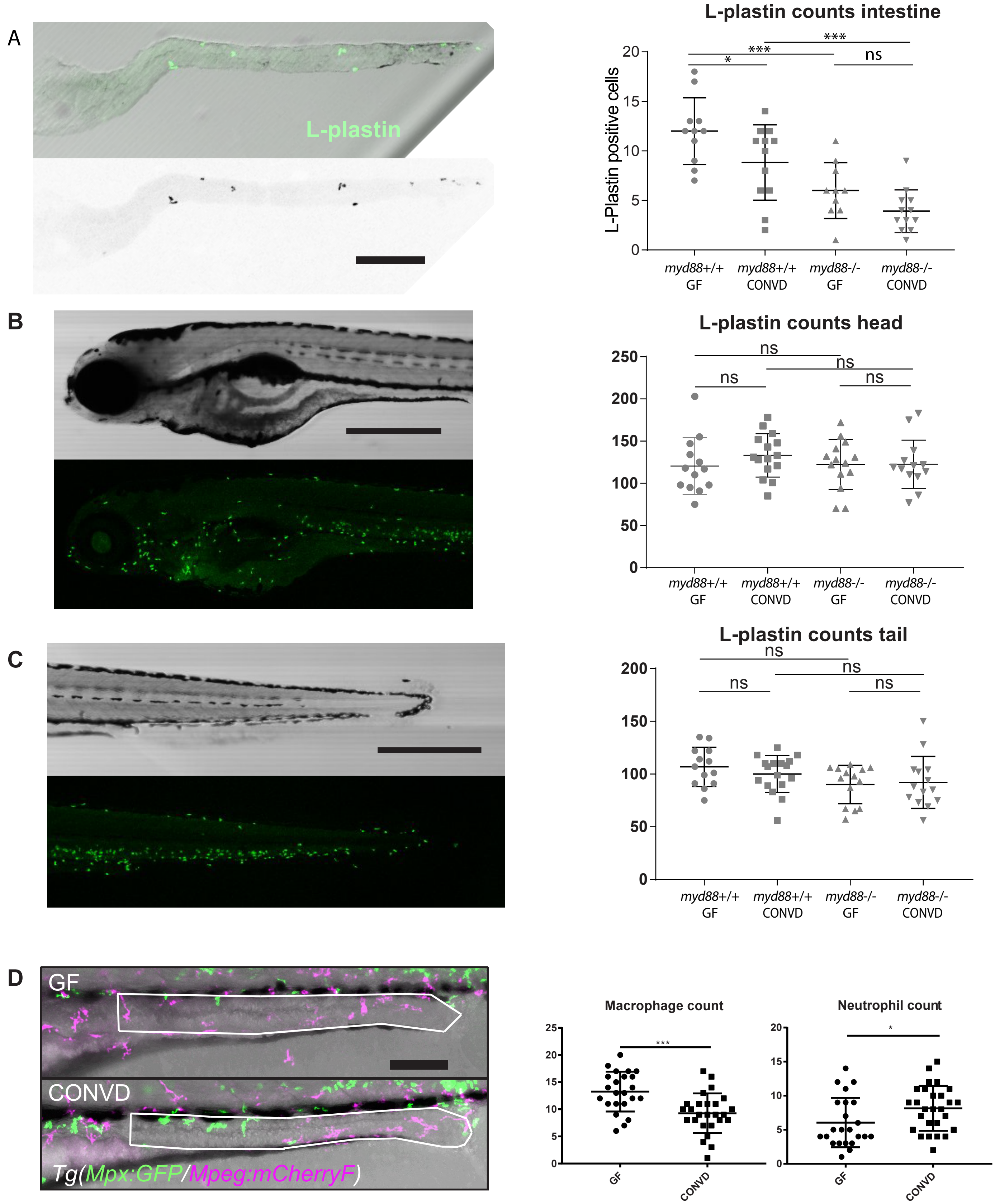

Supplementary Figure 1

(**A**) Excised larval intestines confirm statistically elevated numbers of L-plastin positive cells in germ-free intestines. No significant difference was observed in the intestines of Myd88 deficient larvae. (mean ± s.e.m, n=2 biological replicates, 6-8 embryos per group), *p≤0,05; ***p≤0,001 by two-way ANOVA with Bonferroni correction for multiple comparisons. (**B-C**) Whole embryo assessment of overall leukocyte assessment by L-plastin immunostaining, (mean ± s.e.m, n=2 biological replicates, 6-8 embryos per group) Acquired by confocal microscopy at 5x magnification, no significant differences were observed by two-way ANOVA with Bonferroni correction for multiple comparisons. (**D**) Quantification of macrophages (magenta) and neutrophils (green) in the distal intestine of GF versus CONVD wt larvae of the *Tg(Mpx:GFP/Mpeg:mCherryF)* line at 5 DPF, acquired by confocal microscopy at 20x magnification. The region of analysis is outlined in white lines, (mean ± s.e.m, n=3 biological replicates, 6-8 embryos per group) *p≤0,05; ***p≤0,001 by Student's *t*-test. Scalebars, 200 µm in **A**; 500 µm in **B-C**; 100 µm in **D.**

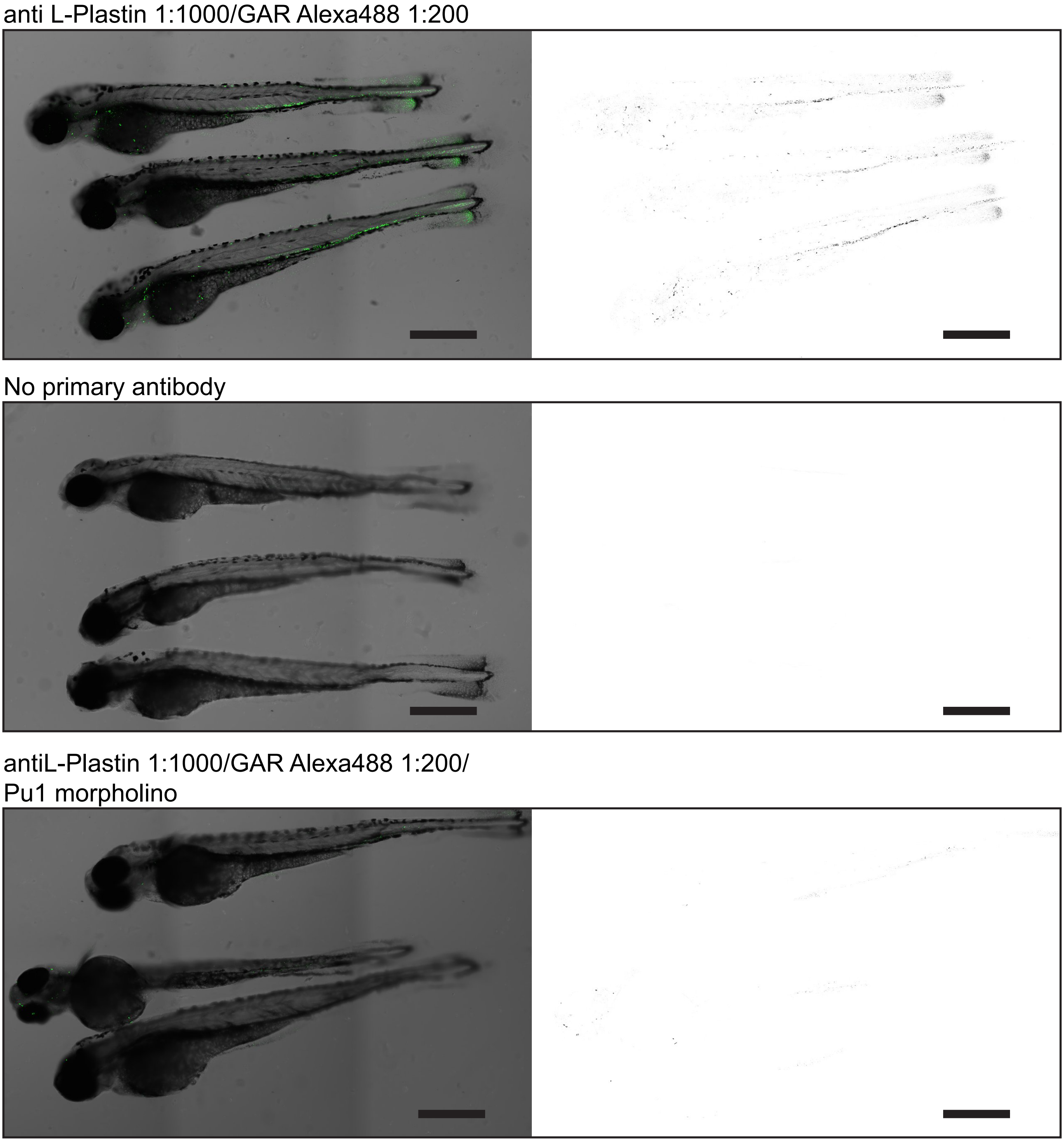

Supplementary Figure 2
Stitched images generated from confocal stacks acquired at 10x magnification, comparing the patterns of L-plastin stain with anti L-plastin primary and goat-anti-rabbit (GAR) second-ary antibody in embryos at 3 DPF to two negative controls; either omitting the primary anti-body or by blocking leukocyte development by injection of pu.1 morpholino[1]. Overlay images on the left, and inverted display of the fluorescent channel on the right. Scalebar is 200 μm.

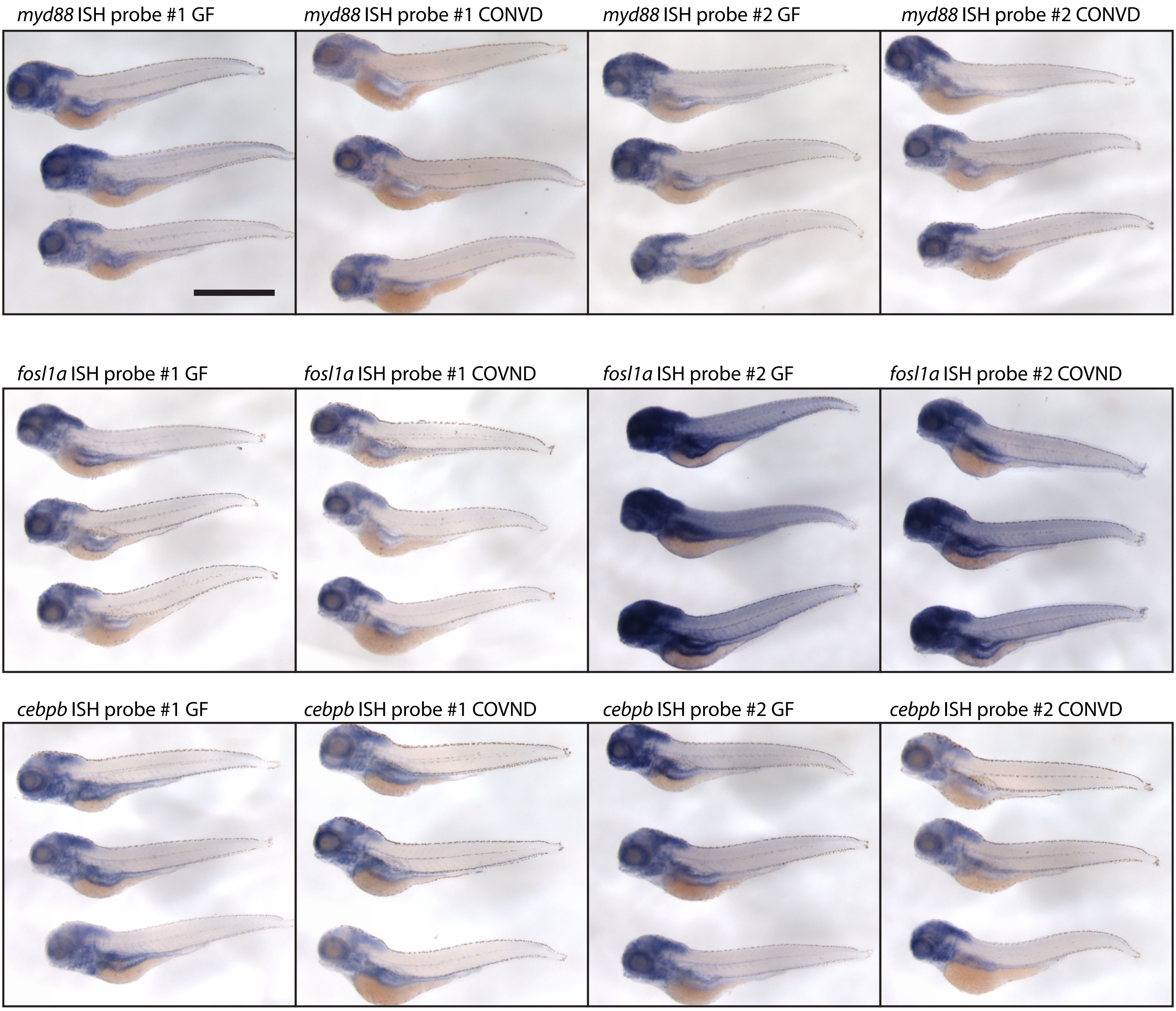

Supplementary Figure 3

Whole-mount in situ hybridization utilizing twon non-overlapping primers targeting *myd88, fosl1a* and *cebpb* in germ-free and conventionalized 5 DPF embryos. Scalebar represents 1 mm.

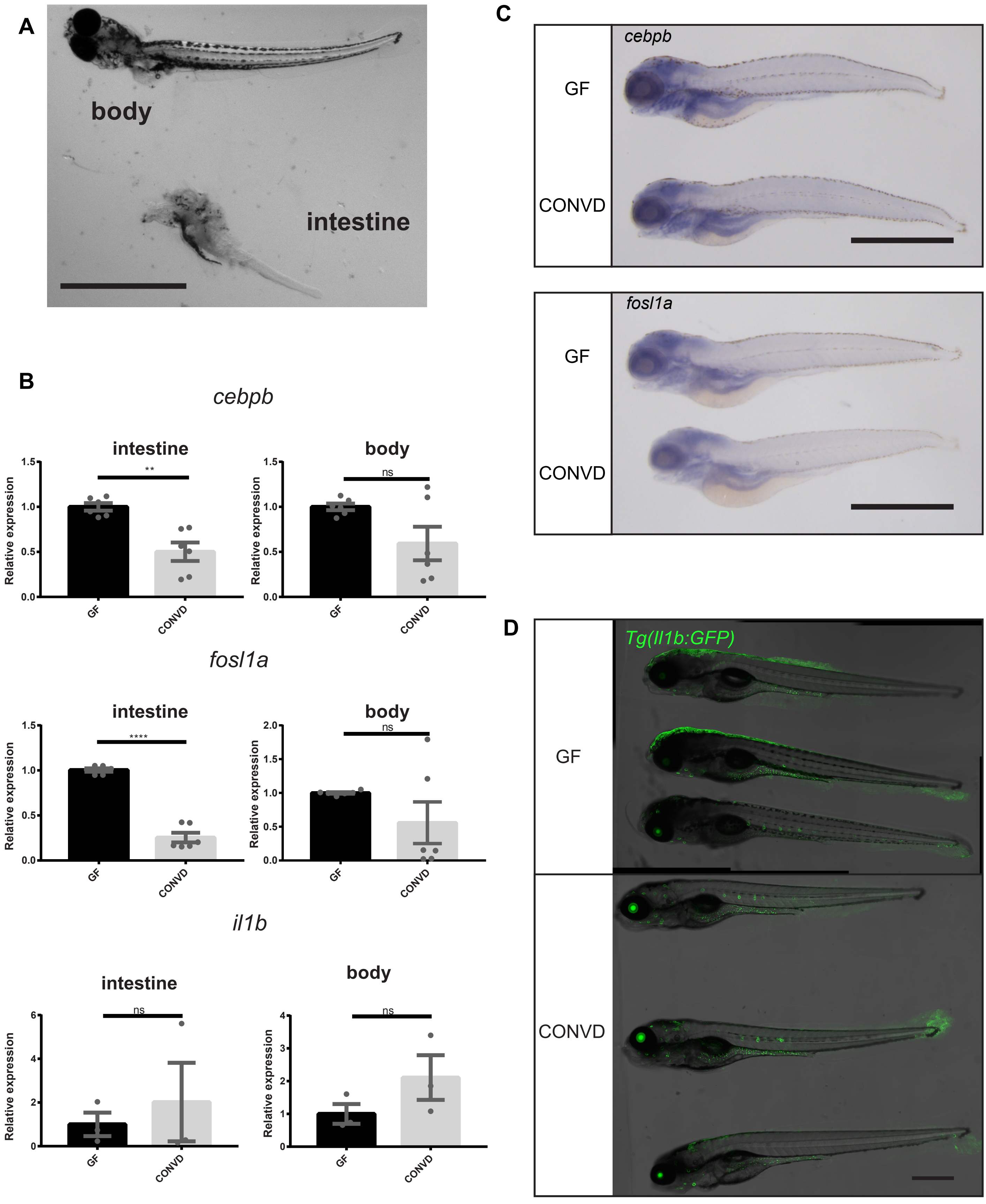

Supplementary Figure 4

(**A**) Image illustrating the separation of intestinal tissues from body tissues, prior to RNA isolation and qPCR analysis of intestinal versus body **(B)** q-PCR analysis comparing the relative expression levels of *cebpb, fosl1a* and *il1b*, in conventionalized (CONVD) versus germ-free (GF) conditions in samples derived from excised intestinal bulp and in the remaining body tissues, (mean ± s.e.m, n=3 biological replicates, 30 embryos per group) **p≤0,01; ****p≤0,0001 by Student's *t*-test **(C)** Whole-mount in sity hybridization results of *cebpb* and *fosl1a,* scale bar represents 1 mm **(D)** Representative images of germ-free and conventionalized larvae in the *Tg(il1b:GFP)* reporter line stitched from confocal stacks acquired with 10x magnification scalebars, 500 µm in **A** and **D**; 1 mm in **C**.

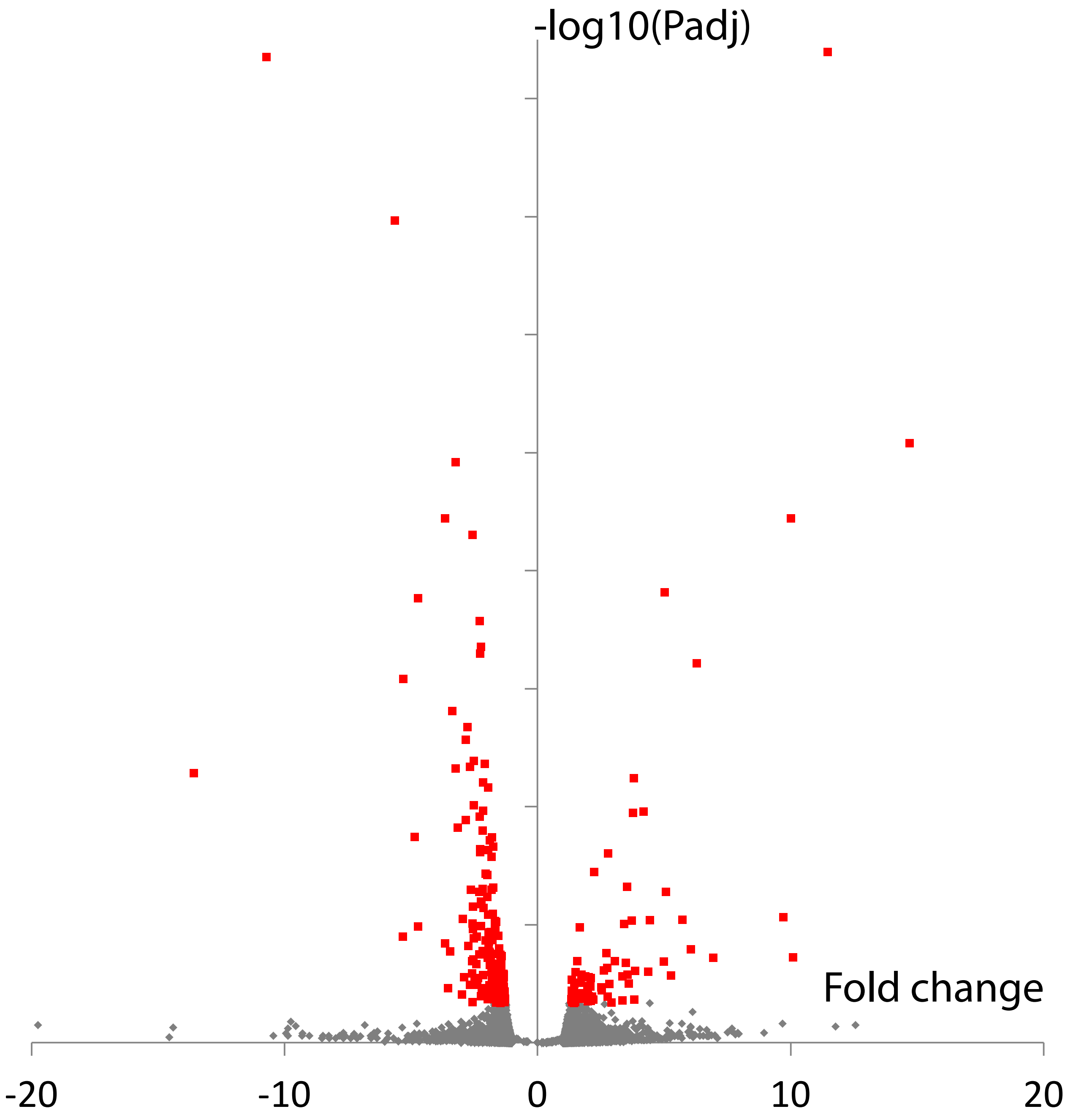

Supplementary Figure 5
Volcano plot showing a graphical representation of the significance cutoff applied to the all transcriptomics data, here given for the transcripts regulated in response to conventionalization. Fold-changes for each transcript was plotted on the X-axis against -log10 transformed Benjamini-Hochberg adjusted p-values on the Y-axis. The transcripts that were considered significant by the two criteria (Foldchange more than +/- 1,3 and adjusted p-value under 0,05) are highlighted in red.

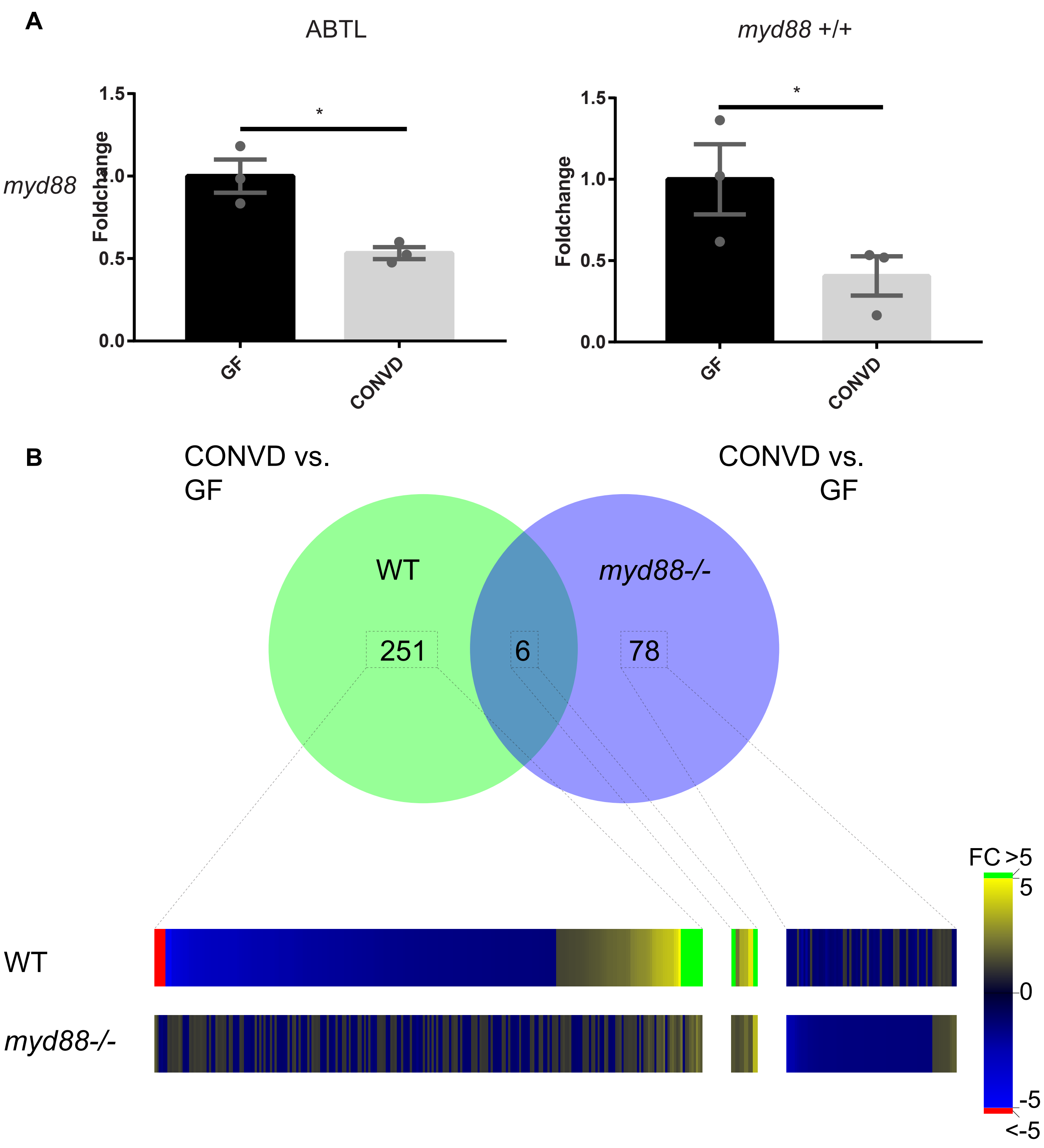

Supplementary Figure 6. Most Transcriptional Alterations Induced by Lacking Colonization are Dependent on Myd88 Signaling

A) qPCR results comparing the *myd88* transcriptional response to conventionalization in WT (ABTL) and the WT (Myd88 proficient) siblings of the *myd88-/-* fish that form the basis of the transcriptomic analysis (mean ± s.e.m, n=3 biological replicates, 15 embryos per group) *p≤0,05 by Student's *t*-test. **B)** Venn diagram showing the overlap between the 257 genes exhibiting differential regulation in germ-free compared to conventionalized groups of WT larvae, and the 84 exhibiting responsiveness in germ-free myd88 mutants compared to conventionalized myd88 mutants. The levels of their regulation in terms of normalized fold changes (FC) are displayed as heat maps underneath.

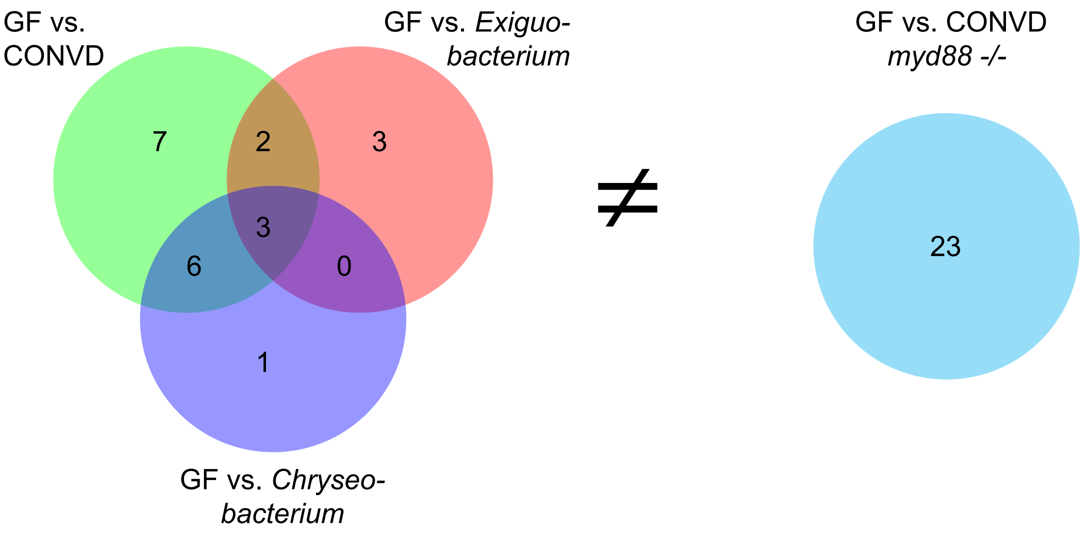

7 GO identifiers were found enriched only in the GF vs CONVD data set

-negative regulation of insulin receptor signaling pathway
-gluconeogenesis
-regulation of keratinocyte differentiation
-glucose 6-phosphate metabolic process
-negative regulation of axon regeneration
-steroid hormone mediated signaling pathway
-retina morphogenesis in camera-type eye

3 GO identifiers were found enriched only in the GF vs *Exiguobacterium* data set

-epiboly involved in gastrulation with mouth forming second
-cell adhesion
-mitochondrial transport

1 GO identifier was found enriched only in the GF vs *Chryseobacterium* data set

-intracellular signal transduction

3 GO identifiers were found enriched in all three WT host data set

-regulation of transcription from RNA polymerase II promoter
-regulation of cell growth
-regulation of transcription, DNA-templated

2 GO identifiers were enriched in GF vs CONVD and GF vs *Exiguoobacterium*

-cell-matrix adhesion
-response to bacterium

6 GO identifiers were enriched in GF vs CONVD and GF vs *Exiguoobacterium*

-negative regulation of cell proliferation
-single organismal cell-cell adhesion
-regulation of MAP kinase activity
-apoptotic process
-activation of cysteine-type endopeptidase activity involved in apoptotic process
-transcription, DNA-templated

≠

23 GO identifiers were found enriched only in the *myd88-/-* background

-lipid transport
-oxidation-reduction process
-reverse cholesterol transport
-cholesterol efflux
-transport
-lipoprotein metabolic process
-regulation of intestinal cholesterol absorption
-positive regulation of fatty acid biosynthetic process
-positive regulation of lipoprotein lipase activity
-phosphatidylcholine metabolic process
-lipid metabolic process
-triglyceride homeostasis
-folic acid-containing compound biosynthetic process
-high-density lipoprotein particle assembly
-very-low-density lipoprotein particle remodeling
-positive regulation of cholesterol esterification
-positive regulation of triglyceride catabolic process
-phospholipid efflux
-cholesterol homeostasis
-neuron projection regeneration
-triglyceride catabolic process
-arachidonic acid metabolic process
-cholesterol biosynthetic process

Supplementary Figure 7. Gene ontology identifiers enriched in different treatments.
Wenn diagram showing the overlap in GO terms that were found enriched in each of the transcriptomics data sets (supplementary table 9). The *myd88-/-* embryos share no GO terms with the WT datasets.

Supplementary Table 1

257 differentially expressed transcripts in response to conventionalization, ranked by Benjamini-Hochberg false discovery rate adjusted p-value.

Rank	Gene-name	Ensembl gene-id	Fold-change CONVD/GF	Padj
1	si:dkey-239b22.1	ENSDARG00000031588	11,46	3,47E-80
2	fosl1a	ENSDARG00000015355	-10,72	4,47E-80
3	si:dkey-79d12.5	ENSDARG00000040284	-5,65	2,03E-66
4	zgc:153932	ENSDARG00000052779	14,70	1,19E-47
5	cebpb	ENSDARG00000042725	-3,25	3,85E-46
6	si:ch211-39f2.3	ENSDARG00000097539	10,01	1,53E-41
7	btg2	ENSDARG00000020298	-3,66	1,53E-41
8	cry1bb	ENSDARG00000091131	-2,58	3,12E-40
9	si:ch211-226h7.3	ENSDARG00000093365	5,02	2,13E-35
10	jdp2b	ENSDARG00000020133	-4,72	5,78E-35
11	sik1	ENSDARG00000058606	-2,29	4,78E-33
12	g6pca.2	ENSDARG00000013721	-2,25	6,92E-31
13	aarsd1	ENSDARG00000015747	-2,27	2,37E-30
14	si:dkey-247k7.2	ENSDARG00000103199	6,29	1,42E-29
15	si:dkey-202l22.6	ENSDARG00000102364	-5,31	2,76E-28
16	batf	ENSDARG00000011818	-3,38	1,33E-25
17	adamts1	ENSDARG00000103519	-2,77	2,91E-24
18	nocta	ENSDARG00000077726	-2,84	3,41E-23
19	ERRFI1 (1 of 2)	ENSDARG00000086098	-2,52	2,00E-21
20	nr4a1	ENSDARG00000000796	-2,09	3,58E-21
21	igfbp1a	ENSDARG00000099351	-2,67	5,61E-21
22	epgn	ENSDARG00000104992	-3,26	7,17E-21
23	5S_rRNA	ENSDARG00000104133	-13,58	1,76E-20
24	si:ch211-207n23.2	ENSDARG00000097157	3,81	4,35E-20
25	odc1	ENSDARG00000007377	-2,16	9,80E-20
26	pnp5a	ENSDARG00000078619	-1,95	2,44E-19
27	si:ch211-192f15.3	ENSDARG00000093331	-2,52	7,92E-18
28	mfsd2ab	ENSDARG00000035909	-2,15	2,21E-17
29	zgc:153932	ENSDARG00000074642	4,20	2,53E-17
30	si:dkeyp-1h4.6	ENSDARG00000093936	3,77	3,03E-17
31	sgk1	ENSDARG00000025522	-2,28	5,99E-17
32	si:dkey-17e16.17	ENSDARG00000091669	-2,85	1,17E-16
33	fosb	ENSDARG00000055751	-3,16	5,26E-16
34	ets2	ENSDARG00000103980	-2,17	8,84E-16
35	plek2	ENSDARG00000012789	-4,86	2,96E-15
36	slc20a1b	ENSDARG00000010641	-1,80	3,15E-15
37	sec23b	ENSDARG00000019360	-1,87	5,68E-15
38	midn	ENSDARG00000018524	-1,74	1,76E-14
39	dusp1	ENSDARG00000100515	-2,26	2,88E-14
40	slmo2	ENSDARG00000009505	-1,96	3,26E-14
41	nr4a3	ENSDARG00000055854	-2,27	4,80E-14
42	zgc:111983	ENSDARG00000006588	2,78	6,38E-14
43	fosl2	ENSDARG00000040623	-1,83	1,21E-13
44	si:ch211-226h7.2	ENSDARG00000094570	2,23	2,18E-12
45	insig1	ENSDARG00000010658	-2,05	2,91E-12
46	stard14	ENSDARG00000031929	-1,99	3,63E-12
47	irg1l	ENSDARG00000062788	3,54	3,73E-11
48	si:dkey-151p11.2	ENSDARG00000102807	-1,75	4,09E-11
49	cldnb	ENSDARG00000009544	-2,17	5,66E-11
50	wu:fc34e06	ENSDARG00000088337	-1,83	5,66E-11
51	kcnk5b	ENSDARG00000012390	-2,62	5,84E-11
52	CU914622.1	ENSDARG00000098256	5,07	8,96E-11
53	fosab	ENSDARG00000031683	-2,31	8,99E-11
54	fosab	ENSDARG00000097234	-1,99	2,18E-10
55	cyp24a1	ENSDARG00000103277	-2,25	5,78E-10
56	slc25a25a	ENSDARG00000010572	-2,22	9,01E-10
57	ptgs2b	ENSDARG00000010276	-2,55	1,46E-09
58	elovl1a	ENSDARG00000099960	-2,13	1,83E-09
59	tuft1a	ENSDARG00000061242	-1,77	6,00E-09
60	cyr61	ENSDARG00000023062	-1,97	6,70E-09
61	si:ch211-182p11.4	ENSDARG00000100106	9,70	1,07E-08
62	klf4	ENSDARG00000079922	-2,96	1,38E-08
63	BX072532.1	ENSDARG00000092704	5,73	1,74E-08
64	mpeg1.2	ENSDARG00000043093	4,44	1,78E-08
65	si:ch211-153b23.3	ENSDARG00000104919	3,72	2,02E-08
66	mid1ip1a	ENSDARG00000041051	-1,68	2,09E-08
67	mmp30	ENSDARG00000045887	-1,64	2,27E-08
68	f3b	ENSDARG00000005673	-2,57	3,33E-08

**69**	CR788316.1	ENSDARG00000073836	3,43	3,79E-08
**70**	rhcga	ENSDARG00000003203	-2,25	5,48E-08
**71**	ponzr4	ENSDARG00000087440	-4,72	5,58E-08
**72**	tmem176l.4	ENSDARG00000074390	1,67	6,78E-08
**73**	ankrd9	ENSDARG00000028804	-1,67	7,58E-08
**74**	dsg2.1	ENSDARG00000076945	-1,69	7,81E-08
**75**	si:dkey-221j11.3	ENSDARG00000093304	-2,55	8,95E-08
**76**	hbegfa	ENSDARG00000075121	-1,87	1,49E-07
**77**	arl5c	ENSDARG00000035719	-1,93	1,65E-07
**78**	sdc4	ENSDARG00000059906	-1,56	3,18E-07
**79**	dok1a	ENSDARG00000078108	-2,41	3,59E-07
**80**	egr2a	ENSDARG00000098502	-5,33	3,59E-07
**81**	bzw1b	ENSDARG00000099148	-2,51	4,99E-07
**82**	elovl6l	ENSDARG00000038639	-1,80	4,99E-07
**83**	crema	ENSDARG00000023217	-1,79	6,91E-07
**84**	cyldb	ENSDARG00000089724	-2,06	7,79E-07
**85**	srsf3a	ENSDARG00000102957	-1,92	8,20E-07
**86**	CU019646.2	ENSDARG00000091234	-3,64	1,34E-06
**87**	egr3	ENSDARG00000089156	-1,95	1,52E-06
**88**	f2rl1.2	ENSDARG00000077540	-2,75	2,01E-06
**89**	tpm4a	ENSDARG00000023963	-1,52	3,15E-06
**90**	si:ch211-226h7.6	ENSDARG00000076196	6,06	3,87E-06
**91**	atf3	ENSDARG00000007823	-2,17	4,87E-06
**92**	egr2a	ENSDARG00000044098	-3,46	5,45E-06
**93**	trib1	ENSDARG00000076142	-1,91	5,97E-06
**94**	arrdc3a	ENSDARG00000052690	-1,89	7,86E-06
**95**	plekhf1	ENSDARG00000102435	2,71	8,08E-06
**96**	ctgfa	ENSDARG00000042934	-1,68	8,37E-06
**97**	si:dkey-11f4.16	ENSDARG00000099799	-2,30	9,22E-06
**98**	mcl1b	ENSDARG00000008363	-1,48	1,02E-05
**99**	chac1	ENSDARG00000070426	-1,42	1,30E-05
**100**	plekhs1	ENSDARG00000079227	10,08	1,60E-05
**101**	slc7a3a	ENSDARG00000020645	-1,44	1,69E-05
**102**	si:ch73-141c7.1	ENSDARG00000009040	-1,98	1,69E-05
**103**	BX005305.6	ENSDARG00000093101	6,93	1,77E-05
**104**	pkp3a	ENSDARG00000051861	-1,45	2,32E-05
**105**	serpine1	ENSDARG00000056795	-2,54	2,38E-05
**106**	socs1a	ENSDARG00000038095	-1,88	3,03E-05
**107**	arrdc3b	ENSDARG00000036028	-2,60	3,08E-05
**108**	mucms1	ENSDARG00000069566	3,05	3,11E-05
**109**	nuak1a	ENSDARG00000020086	-1,87	3,13E-05
**110**	dyrk1aa	ENSDARG00000063570	1,55	3,13E-05
**111**	si:ch211-226h7.5	ENSDARG00000074150	4,98	3,31E-05
**112**	si:dkey-204a24.10	ENSDARG00000036767	3,48	4,60E-05
**113**	ccng2	ENSDARG00000017602	-1,43	4,81E-05
**114**	fkbp5	ENSDARG00000028396	-1,62	4,99E-05
**115**	si:dkey-61d19.4	ENSDARG00000092084	-2,43	5,30E-05
**116**	cdkn1a	ENSDARG00000076554	-1,88	6,55E-05
**117**	ovca2	ENSDARG00000103748	-1,61	1,06E-04
**118**	si:ch211-226h7.4	ENSDARG00000092719	2,75	1,12E-04
**119**	ddit4	ENSDARG00000037618	-1,72	1,43E-04
**120**	muc13a	ENSDARG00000069559	2,62	1,74E-04
**121**	angptl4	ENSDARG00000035859	-1,44	1,80E-04
**122**	si:ch73-181m17.1	ENSDARG00000097080	3,85	1,87E-04
**123**	VGF	ENSDARG00000086665	-1,47	2,26E-04
**124**	BX072532.2	ENSDARG00000094951	4,36	2,28E-04
**125**	slc16a6b	ENSDARG00000060246	-1,83	2,32E-04
**126**	RELN	ENSDARG00000098969	1,50	2,46E-04
**127**	ankrd22	ENSDARG00000002298	-2,59	2,76E-04
**128**	pck1	ENSDARG00000013522	-1,36	3,05E-04
**129**	tob1a	ENSDARG00000032619	-1,35	3,65E-04
**130**	si:ch211-226h7.8	ENSDARG00000095409	3,56	3,74E-04
**131**	si:dkey-162h11.3	ENSDARG00000088432	1,72	3,74E-04
**132**	ucp3	ENSDARG00000091209	-1,35	3,95E-04
**133**	myd88	ENSDARG00000010169	-2,15	4,13E-04
**134**	zgc:174917	ENSDARG00000073820	5,27	4,36E-04
**135**	nmrk2	ENSDARG00000067848	-1,48	4,42E-04
**136**	TAF1C	ENSDARG00000096310	-1,42	4,45E-04
**137**	si:dkey-187j14.4	ENSDARG00000053761	3,35	4,54E-04
**138**	tob1b	ENSDARG00000021372	-1,38	4,69E-04
**139**	si:dkey-187j14.6	ENSDARG00000087424	1,88	4,70E-04
**140**	csrnp1b	ENSDARG00000038429	-1,46	4,70E-04
**141**	zgc:153911	ENSDARG00000002509	-2,90	5,36E-04
**142**	si:dkey-81h8.1	ENSDARG00000094074	-1,34	5,48E-04
**143**	jund	ENSDARG00000067850	-1,43	5,54E-04
**144**	ABCC6	ENSDARG00000094901	-1,81	5,55E-04
**145**	zgc:193505	ENSDARG00000093584	-1,44	5,55E-04
**146**	si:ch211-101l18.8	ENSDARG00000096639	2,02	5,58E-04
**147**	slc22a6l	ENSDARG00000055523	-1,60	5,88E-04

**148**	lye	ENSDARG00000100630	-1,57	5,99E-04
**149**	si:ch211-135f11.1	ENSDARG00000062221	2,09	6,36E-04
**150**	diabloa	ENSDARG00000104172	-2,36	6,47E-04
**151**	usp2b	ENSDARG00000074120	-1,58	6,95E-04
**152**	ucp2	ENSDARG00000043154	-1,36	7,22E-04
**153**	ponzr3	ENSDARG00000088589	-2,48	8,02E-04
**154**	arl14	ENSDARG00000063223	-2,50	8,03E-04
**155**	clk4a	ENSDARG00000089372	1,35	8,06E-04
**156**	rgs13	ENSDARG00000017653	-2,38	8,17E-04
**157**	dio3b	ENSDARG00000095767	-1,57	9,20E-04
**158**	SAT1 (3 of 3)	ENSDARG00000095908	-1,39	9,73E-04
**159**	rgl3a	ENSDARG00000071213	-1,59	9,73E-04
**160**	trib3	ENSDARG00000016200	-1,42	1,04E-03
**161**	nr2e3	ENSDARG00000045904	-1,53	1,11E-03
**162**	dusp2	ENSDARG00000098108	-1,51	1,29E-03
**163**	mbd5	ENSDARG00000059581	1,69	1,35E-03
**164**	nanos1	ENSDARG00000078976	1,58	1,42E-03
**165**	klf18	ENSDARG00000018757	-1,44	1,52E-03
**166**	txnipa	ENSDARG00000036107	-1,60	1,52E-03
**167**	si:dkey-31i7.1	ENSDARG00000102895	3,60	1,63E-03
**168**	PLEKHS1 (3 of 3)	ENSDARG00000090526	2,83	1,68E-03
**169**	aimp2	ENSDARG00000018903	1,44	1,70E-03
**170**	cdh1	ENSDARG00000102750	-1,38	2,02E-03
**171**	pkp2	ENSDARG00000023026	-2,38	2,02E-03
**172**	RGS13	ENSDARG00000102059	-2,67	2,03E-03
**173**	b4galt1	ENSDARG00000002634	-1,51	2,28E-03
**174**	tcap	ENSDARG00000007344	-1,90	2,28E-03
**175**	ehd2b	ENSDARG00000040362	-1,50	2,45E-03
**176**	tmem39a	ENSDARG00000102746	1,95	2,48E-03
**177**	si:ch211-170n20.3	ENSDARG00000087873	-1,38	2,79E-03
**178**	slc25a43	ENSDARG00000102048	2,07	2,79E-03
**179**	anxa2a	ENSDARG00000003216	-1,35	2,95E-03
**180**	b3gnt2b	ENSDARG00000091902	-1,43	3,02E-03
**181**	zgc:113625	ENSDARG00000054160	2,51	3,03E-03
**182**	pmaip1	ENSDARG00000089307	-3,54	3,64E-03
**183**	rgs2	ENSDARG00000098761	-2,06	3,71E-03
**184**	OTUD1	ENSDARG00000100872	-1,79	3,76E-03
**185**	coq10b	ENSDARG00000056141	-1,35	5,09E-03
**186**	pdxp	ENSDARG00000038315	2,53	5,25E-03
**187**	cebpd	ENSDARG00000087303	-1,57	5,53E-03
**188**	nr1d1	ENSDARG00000033160	-1,37	5,69E-03
**189**	lrp6	ENSDARG00000100143	1,43	5,78E-03
**190**	zgc:100868	ENSDARG00000004748	1,34	5,98E-03
**191**	zfand5a	ENSDARG00000018898	-1,31	6,78E-03
**192**	socs3a	ENSDARG00000104400	-2,21	6,80E-03
**193**	epcam	ENSDARG00000040534	-1,35	6,80E-03
**194**	si:ch73-288o11.4	ENSDARG00000097398	-1,63	6,97E-03
**195**	slc3a2b	ENSDARG00000037012	-1,54	7,23E-03
**196**	g6pca.1	ENSDARG00000031616	-1,93	7,62E-03
**197**	rbp7a	ENSDARG00000091906	1,98	7,79E-03
**198**	zgc:154075	ENSDARG00000067630	-1,51	7,96E-03
**199**	slc34a2b	ENSDARG00000036864	-1,48	8,15E-03
**200**	gna14	ENSDARG00000035178	1,77	8,76E-03
**201**	ctgfb	ENSDARG00000104292	-1,48	9,82E-03
**202**	plk2b	ENSDARG00000019130	-1,48	9,88E-03
**203**	sb:cb1058	ENSDARG00000099819	-1,49	1,07E-02
**204**	si:dkey-11f4.14	ENSDARG00000098461	-2,99	1,18E-02
**205**	slc52a3	ENSDARG00000042737	-1,74	1,21E-02
**206**	fam46ba	ENSDARG00000039943	-1,37	1,27E-02
**207**	itpkcb	ENSDARG00000067741	-1,36	1,38E-02
**208**	klf6a	ENSDARG00000029072	-1,32	1,44E-02
**209**	tmprss4b	ENSDARG00000012860	-1,50	1,51E-02
**210**	si:dkey-73p2.2	ENSDARG00000079245	2,02	1,51E-02
**211**	csrnp1a	ENSDARG00000031426	-2,25	1,56E-02
**212**	hsd11b2	ENSDARG00000001975	-1,40	1,59E-02
**213**	CU469570.2	ENSDARG00000101196	2,77	1,59E-02
**214**	grhl3	ENSDARG00000078552	2,15	1,60E-02
**215**	mcm7	ENSDARG00000101180	1,38	1,70E-02
**216**	ca5a	ENSDARG00000101778	-1,36	1,71E-02
**217**	rhcgl1	ENSDARG00000007080	1,84	1,91E-02
**218**	si:dkey-7c18.24	ENSDARG00000041433	1,65	1,96E-02
**219**	ddx24	ENSDARG00000104708	1,48	2,00E-02
**220**	C12H10orf71	ENSDARG00000086945	-1,53	2,02E-02
**221**	vsx1	ENSDARG00000056292	-1,38	2,08E-02
**222**	gpr186	ENSDARG00000094860	-1,52	2,13E-02
**223**	si:ch211-173b8.3	ENSDARG00000103112	-1,46	2,13E-02
**224**	plekhn1	ENSDARG00000087844	-1,77	2,24E-02
**225**	NUP54	ENSDARG00000103296	1,73	2,34E-02
**226**	si:dkey-4i23.7	ENSDARG00000094295	-1,98	2,35E-02

**227**	tefb	ENSDARG00000098103	1,32	2,35E-02
**228**	slc38a4	ENSDARG00000018149	-1,35	2,36E-02
**229**	si:ch211-191a16.2	ENSDARG00000095665	2,19	2,60E-02
**230**	ybx1	ENSDARG00000004757	1,32	2,68E-02
**231**	nfil3-2	ENSDARG00000043237	-1,41	2,72E-02
**232**	si:ch211-219a15.4	ENSDARG00000068951	1,48	2,72E-02
**233**	BRPF1	ENSDARG00000103665	3,82	2,72E-02
**234**	zgc:55512	ENSDARG00000003017	-1,31	2,73E-02
**235**	trim63b	ENSDARG00000086685	-1,30	2,73E-02
**236**	entpd8	ENSDARG00000005565	-1,54	2,74E-02
**237**	foxq1b	ENSDARG00000055395	2,10	2,75E-02
**238**	si:ch73-329n5.6	ENSDARG00000103634	3,34	3,02E-02
**239**	pde4ca	ENSDARG00000013221	-1,38	3,02E-02
**240**	errfi1	ENSDARG00000104039	-1,36	3,04E-02
**241**	si:ch211-261n9.2	ENSDARG00000094165	-1,31	3,10E-02
**242**	elf3	ENSDARG00000077982	2,10	3,24E-02
**243**	fosaa	ENSDARG00000040135	-1,62	3,24E-02
**244**	cplx4c	ENSDARG00000094889	-1,32	3,36E-02
**245**	si:ch211-12p12.4	ENSDARG00000103806	1,98	3,54E-02
**246**	C5H8orf4 (2 of 2)	ENSDARG00000079497	-1,44	3,54E-02
**247**	itga6b	ENSDARG00000069946	-1,31	3,92E-02
**248**	SLC5A10	ENSDARG00000074212	-1,35	3,93E-02
**249**	socs3a	ENSDARG00000025428	1,47	3,93E-02
**250**	ftr36	ENSDARG00000095678	-2,57	3,93E-02
**251**	spry4	ENSDARG00000068732	-1,33	3,95E-02
**252**	C10H11orf30	ENSDARG00000059059	1,37	3,95E-02
**253**	zgc:162183	ENSDARG00000074663	-1,49	4,12E-02
**254**	si:ch211-129c21.1	ENSDARG00000024775	-1,64	4,12E-02
**255**	IRGM (6 of 8)	ENSDARG00000090707	2,92	4,34E-02
**256**	cdk13	ENSDARG00000076791	1,42	4,67E-02
**257**	fhl1b	ENSDARG00000056653	-1,48	4,74E-02

Supplementary Table 2

84 differentially expressed transcripts in response to conventionalization in the *myd88-/-* background, ranked by Benjamini-Hochberg false discovery rate adjusted p-value.

Rank	Gene-name	Ensembl gene-id	Fold-change CONVD/GF	Padj
**1**	nr1d4b	ENSDARG00000059370	-2,22	2,31E-18
**2**	si:ch73-1m18.2	ENSDARG00000096842	-2,21	1,04E-17
**3**	slc15a1b	ENSDARG00000044528	-1,50	7,97E-14
**4**	cyp3a65	ENSDARG00000103295	-1,37	1,31E-12
**5**	apoa4b.2	ENSDARG00000020866	-1,31	8,78E-11
**6**	hpcal1	ENSDARG00000022763	-1,37	2,05E-09
**7**	apobb.1	ENSDARG00000022767	-1,38	2,05E-09
**8**	abca1b	ENSDARG00000079009	-1,38	2,05E-09
**9**	dbpb	ENSDARG00000057652	-1,42	1,36E-08
**11**	mtp	ENSDARG00000008637	-1,36	2,09E-08
**10**	plac8.1	ENSDARG00000043729	-1,37	2,09E-08
**13**	per1a	ENSDARG00000056885	-1,30	3,14E-08
**14**	bhlhe41	ENSDARG00000041691	-1,31	3,14E-08
**12**	ace2	ENSDARG00000016918	-1,33	3,14E-08
**15**	tm4sf4	ENSDARG00000040747	-1,35	4,92E-08
**16**	soul5	ENSDARG00000075015	1,41	2,64E-07
**17**	a1cf	ENSDARG00000002968	-1,38	3,12E-07
**18**	cyp2n13	ENSDARG00000042953	-1,34	4,47E-07
**19**	abcc2	ENSDARG00000014031	-1,43	1,05E-06
**20**	si:ch211-13o20.3	ENSDARG00000091871	-1,86	6,37E-06
**21**	MGAM2	ENSDARG00000101629	-1,71	6,84E-06
**22**	cry5	ENSDARG00000019498	1,34	9,50E-06
**23**	slc6a19a.2	ENSDARG00000091560	-1,40	1,60E-05
**24**	adarb1a	ENSDARG00000041609	1,31	3,36E-05
**25**	si:ch211-93f2.1	ENSDARG00000101726	-1,56	3,36E-05
**26**	slc43a2b	ENSDARG00000061120	-1,32	4,54E-05
**27**	dgat2	ENSDARG00000018846	-1,37	4,76E-05
**28**	BX072532.1	ENSDARG00000092704	3,60	6,17E-05
**29**	cd36	ENSDARG00000032639	-1,31	6,98E-05
**30**	pimr103	ENSDARG00000095525	1,82	8,17E-05
**31**	slc28a1	ENSDARG00000060879	-1,48	1,04E-04
**32**	acsl5	ENSDARG00000075931	-1,35	1,08E-04
**33**	asah2	ENSDARG00000012829	-1,38	1,08E-04
**34**	crtac1a	ENSDARG00000059826	1,66	1,09E-04
**35**	CABZ01041812.1	ENSDARG00000061481	-1,35	1,35E-04
**36**	si:ch73-329n5.6	ENSDARG00000103634	1,59	1,41E-04
**37**	cx28.9	ENSDARG00000041797	-1,36	1,57E-04
**38**	aoc1	ENSDARG00000061355	-1,33	1,98E-04
**39**	apoa4b.1	ENSDARG00000040298	-1,32	3,39E-04
**40**	si:dkey-204a24.10	ENSDARG00000036767	2,11	4,94E-04
**41**	vwa11	ENSDARG00000098805	-1,46	5,42E-04
**42**	cyp2aa8	ENSDARG00000098803	-1,98	5,53E-04
**43**	pimr102	ENSDARG00000086350	1,83	5,99E-04
**44**	sult3st2	ENSDARG00000093619	-1,44	6,16E-04
**45**	si:ch211-89f7.1	ENSDARG00000059729	-1,31	7,64E-04
**46**	lct	ENSDARG00000060671	-1,45	7,68E-04
**47**	PLEKHB1	ENSDARG00000063519	1,42	1,07E-03
**48**	chia.3	ENSDARG00000009612	-1,34	1,20E-03
**49**	nr0b2a	ENSDARG00000044685	-1,44	1,26E-03
**50**	cyp8b1	ENSDARG00000053068	-1,37	1,33E-03
**51**	ugdh	ENSDARG00000019838	-1,31	1,54E-03
**52**	lta4h	ENSDARG00000006029	-1,36	1,56E-03
**53**	serpinh1b	ENSDARG00000019949	1,37	1,75E-03
**54**	cyp2aa9	ENSDARG00000098890	-1,35	1,75E-03
**55**	cyp3c3	ENSDARG00000037873	-1,31	1,96E-03
**56**	zgc:173962	ENSDARG00000039832	-1,32	2,09E-03
**57**	apoa1a	ENSDARG00000012076	-1,33	2,39E-03
**58**	CABZ01088484.1	ENSDARG00000104569	-2,31	3,24E-03
**59**	hpx	ENSDARG00000012609	-2,81	3,58E-03
**60**	si:ch211-226h7.2	ENSDARG00000094570	1,41	4,04E-03
**61**	cipca	ENSDARG00000075397	-1,44	5,98E-03
**62**	slc6a19a.1	ENSDARG00000018621	-1,31	6,37E-03
**63**	nfe2l2b	ENSDARG00000089697	-1,39	8,11E-03
**64**	cpo	ENSDARG00000077688	-1,51	1,03E-02
**65**	CREB3L3 (2 of 2)	ENSDARG00000090903	-1,40	1,06E-02
**66**	zgc:158463	ENSDARG00000089382	-1,76	1,48E-02
**67**	mpeg1.2	ENSDARG00000043093	1,63	1,80E-02
**68**	si:dkeyp-3b12.8	ENSDARG00000103411	-1,71	2,07E-02

**69**	sult3st3	ENSDARG00000042920	-1,34	2,36E-02
**70**	si:ch1073-390k14.1	ENSDARG00000088549	1,30	2,60E-02
**71**	mthfd1a	ENSDARG00000018266	-1,34	2,62E-02
**72**	si:ch211-226h7.3	ENSDARG00000093365	1,63	2,75E-02
**73**	si:ch211-183b15.5	ENSDARG00000097502	1,41	2,89E-02
**74**	otud4	ENSDARG00000099939	-1,67	2,96E-02
**75**	LRAT	ENSDARG00000077652	1,38	2,97E-02
**76**	cyp2p8	ENSDARG00000042982	-1,33	3,04E-02
**77**	sult3st1	ENSDARG00000009885	-1,40	3,13E-02
**78**	ZAN	ENSDARG00000056781	-1,36	3,13E-02
**79**	si:dkey-35h6.1	ENSDARG00000104572	-1,55	3,20E-02
**80**	mthfd1l	ENSDARG00000042221	-1,37	3,42E-02
**81**	slc7a7	ENSDARG00000055226	-1,32	4,49E-02
**82**	pdzd3b	ENSDARG00000055656	-1,43	4,63E-02
**83**	sult3st1	ENSDARG00000103130	-1,38	4,79E-02
**84**	CPN2	ENSDARG00000063518	-1,38	4,89E-02

Supplementary table 3

In the *myd88* deficient background germfree conditions evoke a significant transcriptional induction of a large number of genes related to lipid metabolic processes, reverse cholesterol transport and bile composition. P-values are adjusted to reduce false discovery rates by Benjamini-Hochberg method.

Most of these genes are also differentially regulated in WT versus *myd88* mutants independently of conventionalization status. Genes that meet the significance cut-off criteria ($p_{adj} \leq 0{,}05$, foldchange +/- 1,3) are indicated with (Y)es.

	**Gene**	**Description**	**FC**	**P-adj**	**Differentially regulated in *myd88* mutants versus WT**	
					GF	CONVD
**Lipoprotein assembly**	*a1cf*	apobec1 complementation factor	-1,4	3,10E-07	Y	Y
	abca1b	ATP-binding cassette, sub-family A (ABC1), member 1B	-1,4	2,10E-09	Y	Y
	apoa1a	apolipoprotein A-Ia	-1,3	2,39E-03	Y	N
	apoa4b.1	apolipoprotein A-IV b, tandem duplicate 1	-1,3	3,40E-04	Y	Y
	apoa4b.2	apolipoprotein A-IV b, tandem duplicate 2	-1,3	8,80E-11	Y	N
	apobb.1	apolipoprotein Bb, tandem duplicate 1	-1,4	2,10E-09	Y	Y
	cd36	CD36 molecule (thrombospondin receptor)	-1,3	7,00E-05	Y	Y
	dgat2	diacylglycerol O-acyltransferase 2	-1,4	4,80E-05	Y	Y
	mtp	microsomal triglyceride transfer protein	-1,4	2,10E-08	Y	Y
**Regulation of bile production**	*nr0b2a*	nuclear receptor subfamily 0, group B, member 2a	-1,4	1,26E-03	Y	Y
	cyp8b1	cytochrome P450, family 8, subfamily B, polypeptide 1	-1,4	1,33E-03	Y	Y
	sult3st1	sulfotransferase family 3, cytosolic sulfotransferase 1	-1,4	3,13E-02	Y	Y
	sult3st2	sulfotransferase family 3, cytosolic sulfotransferase 2	-1,4	6,20E-04	Y	Y
	sult3st3	sulfotransferase family 3, cytosolic sulfotransferase 3	-1,3	2,36E-02	Y	Y
**Lipid metabolism**	*lta4h*	leukotriene A4 hydrolase	-1,4	1,56E-03	Y	Y
	acsl5	acyl-CoA synthetase long-chain family member 5	-1,3	1,10E-04	Y	Y
	asah2	N-acylsphingosine amidohydrolase 2	-1,4	1,10E-04	Y	Y

Supplementary Table 4

168 differentially expressed transcripts in response to mono-association with *Chryseobacterium* (ZOR0023), ranked by Benjamini-Hochberg false discovery rate adjusted p-value.

Rank	Gene-name	Ensembl gene-id	Fold-change *Chryseobacterium*/GF	Padj
1	si:dkey-79d12.5	ENSDARG00000040284	-5,93	1,18E-51
2	btg2	ENSDARG00000020298	-3,45	1,18E-51
3	fosl1a	ENSDARG00000015355	-8,16	1,72E-48
4	cyp1a	ENSDARG00000098315	3,69	2,34E-39
5	sik1	ENSDARG00000058606	-2,61	1,88E-33
6	cry1bb	ENSDARG00000091131	-2,54	7,87E-32
7	adamts1	ENSDARG00000103519	-3,50	1,75E-26
8	jdp2b	ENSDARG00000020133	-4,76	1,51E-24
9	nocta	ENSDARG00000077726	-2,73	8,09E-24
10	aarsd1	ENSDARG00000015747	-2,37	7,24E-23
11	g6pca.2	ENSDARG00000013721	-2,37	1,18E-22
12	igfbp1a	ENSDARG00000099351	-2,30	6,15E-19
13	batf	ENSDARG00000011818	-3,46	1,06E-18
14	si:dkey-17e16.17	ENSDARG00000091669	-3,47	2,54E-18
15	ERRFI1 (1 of 2)	ENSDARG00000086098	-2,65	4,18E-18
16	nr4a1	ENSDARG00000000796	-2,11	1,15E-17
17	si:dkey-202l22.6	ENSDARG00000102364	-4,79	6,51E-17
18	sec23b	ENSDARG00000019360	-2,11	2,38E-16
19	5S_rRNA	ENSDARG00000104133	-13,46	1,92E-13
20	rhcga	ENSDARG00000003203	-3,91	1,30E-12
21	ets2	ENSDARG00000103980	-2,25	1,90E-12
22	ponzr4	ENSDARG00000087440	-9,20	1,91E-12
23	dusp1	ENSDARG00000100515	-2,07	3,88E-12
24	epgn	ENSDARG00000104992	-2,83	1,43E-11
25	fosab	ENSDARG00000031683	-1,94	1,69E-11
26	cebpb	ENSDARG00000042725	-2,72	1,95E-11
27	midn	ENSDARG00000018524	-1,72	3,18E-11
28	si:ch211-170n20.3	ENSDARG00000087873	3,07	3,26E-11
29	sgk1	ENSDARG00000025522	-2,00	5,49E-11
30	fosl2	ENSDARG00000040623	-1,83	1,98E-10
31	mfsd2ab	ENSDARG00000035909	-1,94	6,09E-10
32	cyldb	ENSDARG00000089724	-3,25	6,39E-10
33	fosb	ENSDARG00000055751	-3,08	8,50E-10
34	cyr61	ENSDARG00000023062	-1,95	2,06E-09
35	wu:fc34e06	ENSDARG00000088337	-1,95	2,84E-09
36	pnp5a	ENSDARG00000078619	-1,87	3,61E-09
37	cyp24a1	ENSDARG00000103277	-1,98	1,84E-08
38	plek2	ENSDARG00000012789	-3,43	2,53E-08
39	mmp30	ENSDARG00000045887	-1,74	4,10E-08
40	ponzr3	ENSDARG00000088589	-4,04	4,21E-08
41	tuft1a	ENSDARG00000061242	-1,85	4,23E-08
42	slc25a25a	ENSDARG00000010572	-2,35	4,87E-08
43	si:ch211-207n23.2	ENSDARG00000097157	2,75	6,41E-08
44	si:dkey-221j11.3	ENSDARG00000093304	-2,82	2,13E-07
45	si:ch211-101l18.8	ENSDARG00000096639	2,76	2,16E-07
46	dsg2.1	ENSDARG00000076945	-1,73	7,27E-07
47	elovl1a	ENSDARG00000099960	-2,20	2,04E-06
48	elovl6l	ENSDARG00000038639	-2,06	2,04E-06
49	si:dkey-151p11.2	ENSDARG00000102807	-1,67	2,19E-06
50	CU019646.2	ENSDARG00000091234	-3,23	2,79E-06
51	jund	ENSDARG00000067850	-1,67	2,96E-06
52	si:dkey-247k7.2	ENSDARG00000103199	4,54	3,09E-06
53	ankrd9	ENSDARG00000028804	-1,76	3,32E-06
54	stard14	ENSDARG00000031929	-1,76	3,32E-06
55	cdkn1a	ENSDARG00000076554	-2,36	3,41E-06
56	crema	ENSDARG00000023217	-1,88	8,08E-06
57	rgs2	ENSDARG00000098761	-2,64	8,08E-06
58	klf4	ENSDARG00000079922	-3,47	1,49E-05
59	arrdc3a	ENSDARG00000052690	-1,87	1,63E-05
60	kcnk5b	ENSDARG00000012390	-2,25	1,63E-05
61	slc20a1b	ENSDARG00000010641	-1,65	2,13E-05
62	dok1a	ENSDARG00000078108	-2,63	2,72E-05
63	srsf3a	ENSDARG00000102957	-2,01	2,93E-05
64	f3b	ENSDARG00000005673	-2,40	3,09E-05
65	insig1	ENSDARG00000010658	-1,78	3,55E-05

**66**	slc7a3a	ENSDARG00000020645	-1,47	3,85E-05
**67**	mcl1b	ENSDARG00000008363	-1,54	4,01E-05
**68**	cebpd	ENSDARG00000087303	-1,78	7,39E-05
**69**	foxq1a	ENSDARG00000030896	2,55	8,13E-05
**70**	arl5c	ENSDARG00000035719	-2,06	9,80E-05
**71**	fkbp5	ENSDARG00000028396	-1,61	1,51E-04
**72**	tob1b	ENSDARG00000021372	-1,47	1,83E-04
**73**	slmo2	ENSDARG00000009505	-1,62	1,84E-04
**74**	pkhd1l1	ENSDARG00000091116	1,78	1,84E-04
**75**	csrnp1b	ENSDARG00000038429	-1,63	1,87E-04
**76**	ddit4	ENSDARG00000037618	-1,73	2,27E-04
**77**	dusp2	ENSDARG00000098108	-1,64	2,37E-04
**78**	sb:cb1058	ENSDARG00000099819	-1,65	2,80E-04
**79**	fosab	ENSDARG00000097234	-1,78	3,05E-04
**80**	f2rl1.2	ENSDARG00000077540	-2,81	3,22E-04
**81**	nuak1a	ENSDARG00000020086	-2,03	3,30E-04
**82**	nr4a3	ENSDARG00000055854	-1,86	3,80E-04
**83**	si:ch211-192f15.3	ENSDARG00000093331	-2,03	3,92E-04
**84**	socs1a	ENSDARG00000038095	-2,14	4,46E-04
**85**	ptgs2b	ENSDARG00000010276	-2,11	5,73E-04
**86**	si:dkey-61d19.4	ENSDARG00000092084	-2,38	6,77E-04
**87**	elf3	ENSDARG00000077982	1,95	7,62E-04
**88**	pkp2	ENSDARG00000023026	-2,51	7,62E-04
**89**	slc3a2b	ENSDARG00000037012	-1,66	8,49E-04
**90**	gadd45ab	ENSDARG00000104571	-1,64	1,12E-03
**91**	klf18	ENSDARG00000018757	-1,74	1,19E-03
**92**	egr2a	ENSDARG00000098502	-4,23	1,24E-03
**93**	slc25a43	ENSDARG00000102048	-1,53	1,45E-03
**94**	csrnp1a	ENSDARG00000031426	-2,79	1,48E-03
**95**	atf3	ENSDARG00000007823	-1,91	1,53E-03
**96**	klf9	ENSDARG00000068194	-1,64	1,76E-03
**97**	ccng2	ENSDARG00000017602	-1,44	1,77E-03
**98**	sdc4	ENSDARG00000059906	-1,56	1,78E-03
**99**	OTUD1	ENSDARG00000100872	-2,23	1,89E-03
**100**	pkp3a	ENSDARG00000051861	-1,40	1,89E-03
**101**	trib1	ENSDARG00000076142	-1,79	1,93E-03
**102**	tob1a	ENSDARG00000032619	-1,46	1,94E-03
**103**	bzw1b	ENSDARG00000099148	-2,21	1,98E-03
**104**	zfand5a	ENSDARG00000018898	-1,43	2,00E-03
**105**	egr3	ENSDARG00000089156	-1,84	2,00E-03
**106**	nr2e3	ENSDARG00000045904	-1,69	2,31E-03
**107**	socs3a	ENSDARG00000104400	-2,02	2,31E-03
**108**	cldnb	ENSDARG00000009544	-1,99	2,69E-03
**109**	stx11b.1	ENSDARG00000029290	-1,80	2,75E-03
**110**	chac1	ENSDARG00000070426	-1,39	2,88E-03
**111**	plk2b	ENSDARG00000019130	-1,57	3,95E-03
**112**	ier2	ENSDARG00000099195	-1,58	3,95E-03
**113**	zgc:153932	ENSDARG00000052779	4,81	3,96E-03
**114**	mid1ip1a	ENSDARG00000041051	-1,48	3,97E-03
**115**	rgl3a	ENSDARG00000071213	-1,75	3,97E-03
**116**	krt15	ENSDARG00000036840	-1,50	4,14E-03
**117**	egr2a	ENSDARG00000044098	-3,30	4,15E-03
**118**	si:ch73-141c7.1	ENSDARG00000009040	-1,90	4,90E-03
**119**	si:dkey-11f4.16	ENSDARG00000099799	-2,08	5,27E-03
**120**	si:ch211-261n9.2	ENSDARG00000094165	-1,44	5,49E-03
**121**	foxq1b	ENSDARG00000055395	2,54	6,02E-03
**122**	rgs13	ENSDARG00000017653	-2,89	6,30E-03
**123**	zgc:154075	ENSDARG00000067630	-1,74	7,14E-03
**124**	rhcgl1	ENSDARG00000007080	2,27	7,64E-03
**125**	pfkfb4b	ENSDARG00000029075	-1,37	7,64E-03
**126**	hsd11b2	ENSDARG00000001975	-1,48	7,69E-03
**127**	nfil3-2	ENSDARG00000043237	-1,45	7,71E-03
**128**	myd88	ENSDARG00000010169	-1,90	8,82E-03
**129**	si:ch211-12p12.4	ENSDARG00000103806	2,30	8,93E-03
**130**	capn3b	ENSDARG00000043035	1,67	1,03E-02
**131**	clk4a	ENSDARG00000089372	1,35	1,25E-02
**132**	adgrb1b	ENSDARG00000078529	-1,38	1,25E-02
**133**	si:ch73-288o11.4	ENSDARG00000097398	-1,73	1,28E-02
**134**	TAF1C	ENSDARG00000096310	-1,39	1,36E-02
**135**	b4galt1	ENSDARG00000002634	-1,58	1,36E-02
**136**	trim35-12	ENSDARG00000101264	-2,00	1,36E-02
**137**	VGF	ENSDARG00000086665	-1,40	1,42E-02
**138**	zgc:162183	ENSDARG00000074663	-1,63	1,42E-02
**139**	cdh1	ENSDARG00000102750	-1,37	1,47E-02
**140**	spry4	ENSDARG00000068732	-1,44	1,47E-02
**141**	KRT80	ENSDARG00000056248	-1,32	1,58E-02
**142**	pmaip1	ENSDARG00000089307	-3,24	1,73E-02
**143**	zgc:193505	ENSDARG00000093584	-1,43	1,85E-02
**144**	trib3	ENSDARG00000016200	-1,40	1,85E-02

**145**	nanos1	ENSDARG00000078976	1,51	2,20E-02
**146**	ankrd22	ENSDARG00000002298	-2,27	2,35E-02
**147**	klf3	ENSDARG00000015495	-1,33	2,49E-02
**148**	ctgfa	ENSDARG00000042934	-1,46	2,64E-02
**149**	arrdc3b	ENSDARG00000036028	-2,26	2,70E-02
**150**	hbegfa	ENSDARG00000075121	-1,55	2,70E-02
**151**	zgc:153932	ENSDARG00000074642	2,12	2,79E-02
**152**	ABCC6	ENSDARG00000094901	-2,00	2,79E-02
**153**	plekhn1	ENSDARG00000087844	-1,92	2,79E-02
**154**	larp1	ENSDARG00000101722	1,39	2,79E-02
**155**	CABZ01103755.1	ENSDARG00000099702	5,07	2,86E-02
**156**	RELN	ENSDARG00000098969	1,38	3,09E-02
**157**	mknk1	ENSDARG00000018411	-1,57	3,19E-02
**158**	diabloa	ENSDARG00000104172	-2,47	3,25E-02
**159**	ovca2	ENSDARG00000103748	-1,43	3,26E-02
**160**	klf6a	ENSDARG00000029072	-1,34	3,49E-02
**161**	si:ch211-285j22.3	ENSDARG00000100703	2,22	3,55E-02
**162**	ptgdsb	ENSDARG00000027088	1,34	3,71E-02
**163**	lama3	ENSDARG00000071462	-1,73	3,93E-02
**164**	frmd4bb	ENSDARG00000076496	-1,62	4,03E-02
**165**	tpm4a	ENSDARG00000023963	-1,42	4,52E-02
**166**	txnipa	ENSDARG00000036107	-1,50	4,73E-02
**167**	fam46ba	ENSDARG00000039943	-1,35	4,73E-02
**168**	odc1	ENSDARG00000007377	-1,92	4,93E-02

Supplementary Table 5

122 differentially expressed transcripts in response to mono-association with *Exiguobacterium* (ZWU0009), ranked by Benjamini-Hochberg false discovery rate adjusted p-value.

Rank	Gene-name	Ensembl gene-id	Fold-change *Exiguobacterium/GF*	P-adj
1	zgc:153932	ENSDARG00000052779	9,66	2,33E-39
2	fosl1a	ENSDARG00000015355	-4,09	3,27E-32
3	zgc:111983	ENSDARG00000006588	2,62	3,31E-32
4	odc1	ENSDARG00000007377	-2,45	2,60E-26
5	si:ch211-226h7.3	ENSDARG00000093365	3,94	6,68E-26
6	si:dkey-239b22.1	ENSDARG00000031588	8,37	1,17E-21
7	mpeg1.2	ENSDARG00000043093	4,99	1,30E-19
8	si:dkey-79d12.5	ENSDARG00000040284	-2,66	6,09E-17
9	si:ch211-39f2.3	ENSDARG00000097539	5,88	6,09E-17
10	slc20a1b	ENSDARG00000010641	-1,73	3,70E-16
11	irg1l	ENSDARG00000062788	4,89	3,69E-13
12	plekhs1	ENSDARG00000079227	11,10	6,54E-12
13	si:dkey-202l22.6	ENSDARG00000102364	-2,88	1,68E-11
14	midn	ENSDARG00000018524	-1,62	3,22E-11
15	zgc:153932	ENSDARG00000074642	3,12	3,22E-11
16	nr4a3	ENSDARG00000055854	-2,16	7,29E-11
17	dsg2.1	ENSDARG00000076945	-1,85	1,50E-10
18	plek2	ENSDARG00000012789	-3,81	8,72E-10
19	slmo2	ENSDARG00000009505	-1,69	2,96E-09
20	si:ch211-192f15.3	ENSDARG00000093331	-2,18	2,68E-08
21	cebpb	ENSDARG00000042725	-1,69	2,88E-08
22	adamts1	ENSDARG00000103519	-2,08	3,51E-08
23	BX072532.1	ENSDARG00000092704	4,99	3,84E-08
24	klf4	ENSDARG00000079922	-3,03	4,00E-08
25	si:dkey-247k7.2	ENSDARG00000103199	4,47	4,41E-08
26	mmp30	ENSDARG00000045887	-1,63	7,50E-08
27	si:ch211-153b23.3	ENSDARG00000104919	6,00	1,04E-07
28	fosl2	ENSDARG00000040623	-1,57	1,12E-07
29	5S_rRNA	ENSDARG00000104133	-4,54	2,08E-07
30	mfsd2ab	ENSDARG00000035909	-1,83	3,20E-07
31	clk4a	ENSDARG00000089372	1,45	4,05E-07
32	slc16a6b	ENSDARG00000060246	-2,20	6,30E-07
33	ets2	ENSDARG00000103980	-1,72	8,71E-07
34	kcnk5b	ENSDARG00000012390	-2,17	1,00E-06
35	si:dkey-151p11.2	ENSDARG00000102807	-1,55	1,69E-06
36	tuft1a	ENSDARG00000061242	-1,68	1,91E-06
37	elovl1a	ENSDARG00000099960	-1,95	3,28E-06
38	ucp2	ENSDARG00000043154	-1,43	3,71E-06
39	cldnb	ENSDARG00000009544	-1,78	4,72E-06
40	tmem176l.4	ENSDARG00000074390	1,59	5,14E-06
41	nr4a1	ENSDARG00000000796	-1,49	6,78E-06
42	pkp3a	ENSDARG00000051861	-1,47	7,41E-06
43	arrdc3b	ENSDARG00000036028	-2,80	1,04E-05
44	nocta	ENSDARG00000077726	-1,93	1,81E-05
45	fosb	ENSDARG00000055751	-2,00	3,18E-05
46	slc7a3a	ENSDARG00000020645	-1,42	3,73E-05
47	ucp3	ENSDARG00000091209	-1,38	3,98E-05
48	dok1a	ENSDARG00000078108	-2,37	4,63E-05
49	si:ch211-226h7.2	ENSDARG00000094570	2,03	5,08E-05
50	angptl4	ENSDARG00000035859	-1,46	5,35E-05
51	si:ch73-329n5.6	ENSDARG00000103634	3,09	5,67E-05
52	jdp2b	ENSDARG00000020133	-1,79	6,40E-05
53	tpm4a	ENSDARG00000023963	-1,48	6,40E-05
54	ERRFI1 (1 of 2)	ENSDARG00000086098	-1,78	6,68E-05
55	insig1	ENSDARG00000010658	-1,64	6,78E-05
56	PLEKHS1 (3 of 3)	ENSDARG00000090526	3,22	1,07E-04
57	arrdc3a	ENSDARG00000052690	-1,81	1,67E-04
58	ccl20a.3	ENSDARG00000101040	32,86	1,77E-04
59	igfbp1a	ENSDARG00000099351	-1,77	1,87E-04
60	slc25a25a	ENSDARG00000010572	-1,66	1,87E-04
61	bzw1b	ENSDARG00000099148	-2,08	1,87E-04
62	CU914622.1	ENSDARG00000098256	3,50	2,44E-04
63	si:dkey-30j22.1	ENSDARG00000042829	1,61	3,18E-04
64	si:ch211-226h7.6	ENSDARG00000076196	5,57	4,37E-04
65	pnp5a	ENSDARG00000078619	-1,50	4,94E-04
66	si:dkey-187j14.4	ENSDARG00000053761	3,50	4,94E-04

**67**	CU019646.2	ENSDARG00000091234	-2,11	6,61E-04
**68**	sdc4	ENSDARG00000059906	-1,36	7,72E-04
**69**	higd1a	ENSDARG00000022303	-1,53	7,94E-04
**70**	f3b	ENSDARG00000005673	-2,05	8,73E-04
**71**	cdh1	ENSDARG00000102750	-1,41	8,73E-04
**72**	zgc:100868	ENSDARG00000004748	1,38	9,21E-04
**73**	si:dkey-204a24.10	ENSDARG00000036767	2,09	1,19E-03
**74**	cyp24a1	ENSDARG00000103277	-2,01	1,26E-03
**75**	sgk1	ENSDARG00000025522	-1,59	1,37E-03
**76**	batf	ENSDARG00000011818	-1,62	1,54E-03
**77**	si:dkey-11f4.16	ENSDARG00000099799	-1,88	1,86E-03
**78**	si:ch211-153b23.5	ENSDARG00000058206	1,61	2,29E-03
**79**	SAT1 (3 of 3)	ENSDARG00000095908	-1,41	2,58E-03
**80**	si:ch211-191a16.5	ENSDARG00000096545	1,83	2,77E-03
**81**	crema	ENSDARG00000023217	-1,53	3,17E-03
**82**	muc13a	ENSDARG00000069559	2,18	3,23E-03
**83**	itga6b	ENSDARG00000069946	-1,46	3,23E-03
**84**	si:dkey-31i7.1	ENSDARG00000102895	3,73	4,01E-03
**85**	si:dkey-61d19.4	ENSDARG00000092084	-2,06	4,53E-03
**86**	SLC5A10	ENSDARG00000074212	-1,44	4,53E-03
**87**	BX005305.6	ENSDARG00000093101	5,83	4,53E-03
**88**	si:ch211-226h7.4	ENSDARG00000092719	2,51	4,97E-03
**89**	adgrb1b	ENSDARG00000078529	-1,36	5,55E-03
**90**	myd88	ENSDARG00000010169	-1,89	5,59E-03
**91**	si:dkey-175d9.2	ENSDARG00000093476	3,40	6,41E-03
**92**	fgf19	ENSDARG00000098663	2,03	7,79E-03
**93**	anxa2a	ENSDARG00000003216	-1,34	8,51E-03
**94**	coq10b	ENSDARG00000056141	-1,34	9,07E-03
**95**	sik1	ENSDARG00000058606	-1,33	1,01E-02
**96**	ctgfa	ENSDARG00000042934	-1,43	1,01E-02
**97**	b3gnt2b	ENSDARG00000091902	-1,42	1,05E-02
**98**	lye	ENSDARG00000100630	-1,46	1,19E-02
**99**	ddx5	ENSDARG00000038068	-1,33	1,22E-02
**100**	si:ch211-101l18.8	ENSDARG00000096639	1,92	1,39E-02
**101**	g6pca.1	ENSDARG00000031616	-2,13	1,45E-02
**102**	zgc:174917	ENSDARG00000073820	4,49	1,49E-02
**103**	wu:fc34e06	ENSDARG00000088337	-1,54	1,59E-02
**104**	csrnp1a	ENSDARG00000031426	-2,34	1,73E-02
**105**	ehd2b	ENSDARG00000040362	-1,41	1,79E-02
**106**	si:ch211-226h7.8	ENSDARG00000095409	3,23	1,80E-02
**107**	egr1	ENSDARG00000037421	1,51	2,28E-02
**108**	BX072532.2	ENSDARG00000094951	3,52	2,32E-02
**109**	serpine1	ENSDARG00000056795	-1,75	2,98E-02
**110**	si:ch211-226h7.5	ENSDARG00000074150	4,74	2,98E-02
**111**	RGS13	ENSDARG00000102059	-2,43	3,06E-02
**112**	atf3	ENSDARG00000007823	-1,62	3,41E-02
**113**	rgs13	ENSDARG00000017653	-2,43	3,56E-02
**114**	klf11b	ENSDARG00000013794	-1,44	3,92E-02
**115**	wu:fj16a03	ENSDARG00000100952	-1,46	4,08E-02
**116**	si:ch211-191a16.2	ENSDARG00000095665	2,25	4,15E-02
**117**	dsc2l	ENSDARG00000039677	-1,34	4,18E-02
**118**	cry1bb	ENSDARG00000091131	-1,45	4,37E-02
**119**	ABCC6	ENSDARG00000094901	-1,73	4,37E-02
**120**	si:dkey-187j14.6	ENSDARG00000087424	1,69	4,37E-02
**121**	si:ch211-182p11.4	ENSDARG00000100106	5,38	4,76E-02
**122**	fkbp5	ENSDARG00000028396	-1,51	4,92E-02

Supplementary Table 6

65 transcripts were differentially expressed under germ-free conditions compared to either conventionalized or the two monoassociated treatment groups.

Gene-name	Ensembl gene-id
clk4a	ENSDARG00000089372
sik1	ENSDARG00000058606
cry1bb	ENSDARG00000091131
nr4a1	ENSDARG00000000796
si:dkey-151p11.2	ENSDARG00000102807
insig1	ENSDARG00000010658
zgc:153932	ENSDARG00000074642
si:dkey-247k7.2	ENSDARG00000103199
zgc:153932	ENSDARG00000052779
arrdc3a	ENSDARG00000052690
slc20a1b	ENSDARG00000010641
nocta	ENSDARG00000077726
midn	ENSDARG00000018524
adamts1	ENSDARG00000103519
ctgfa	ENSDARG00000042934
dsg2.1	ENSDARG00000076945
fosl1a	ENSDARG00000015355
odc1	ENSDARG00000007377
si:dkey-79d12.5	ENSDARG00000040284
si:dkey-202l22.6	ENSDARG00000102364
nr4a3	ENSDARG00000055854
plek2	ENSDARG00000012789
slmo2	ENSDARG00000009505
si:ch211-192f15.3	ENSDARG00000093331
cebpb	ENSDARG00000042725
klf4	ENSDARG00000079922
mmp30	ENSDARG00000045887
fosl2	ENSDARG00000040623
5S_rRNA	ENSDARG00000104133
mfsd2ab	ENSDARG00000035909
ets2	ENSDARG00000103980
kcnk5b	ENSDARG00000012390
tuft1a	ENSDARG00000061242
elovl1a	ENSDARG00000099960
cldnb	ENSDARG00000009544
pkp3a	ENSDARG00000051861
arrdc3b	ENSDARG00000036028
fosb	ENSDARG00000055751
slc7a3a	ENSDARG00000020645
dok1a	ENSDARG00000078108
jdp2b	ENSDARG00000020133
tpm4a	ENSDARG00000023963
ERRFI1 (1 of 2)	ENSDARG00000086098
igfbp1a	ENSDARG00000099351
slc25a25a	ENSDARG00000010572
bzw1b	ENSDARG00000099148
pnp5a	ENSDARG00000078619
CU019646.2	ENSDARG00000091234
sdc4	ENSDARG00000059906
f3b	ENSDARG00000005673
cdh1	ENSDARG00000102750
cyp24a1	ENSDARG00000103277
sgk1	ENSDARG00000025522
batf	ENSDARG00000011818
si:dkey-11f4.16	ENSDARG00000099799
crema	ENSDARG00000023217
si:dkey-61d19.4	ENSDARG00000092084
myd88	ENSDARG00000010169
si:ch211-101l18.8	ENSDARG00000096639
wu:fc34e06	ENSDARG00000088337
csrnp1a	ENSDARG00000031426
atf3	ENSDARG00000007823
rgs13	ENSDARG00000017653
ABCC6	ENSDARG00000094901
fkbp5	ENSDARG00000028396

Supplementary Table 7

Gene ontology analysis for each set of statistically regulated transcripts in the study. Listing for each GO term: the number of genes out of the set, the percentage of the total set and the fold enrichment, and ranked by p-value.

GO analysis of 257 transcripts differentially expressed after conventionalization compared to germ-free (see supplementary table 1).

Rank	GO Term	Number of genes	Percentage of total	P-value	Fold enrichment
1	regulation of transcription from RNA polymerase II promoter	13	5.882353	2.19E-05	4.671679
2	regulation of cell growth	5	2.262443	6.96E-04	12.2551
3	regulation of transcription, DNA-templated	27	12.21719	9.82E-04	1.97017
4	cell-matrix adhesion	5	2.262443	0.001305	10.39019
5	negative regulation of insulin receptor signaling pathway	3	1.357466	0.004573	28.67692
6	negative regulation of cell proliferation	4	1.809955	0.01136	8.496866
7	single organismal cell-cell adhesion	4	1.809955	0.014312	7.803244
8	gluconeogenesis	3	1.357466	0.016348	15.09312
9	regulation of keratinocyte differentiation	2	0.904977	0.020681	95.58974
10	regulation of MAP kinase activity	2	0.904977	0.030862	63.7265
11	apoptotic process	5	2.262443	0.039422	3.885762
12	glucose 6-phosphate metabolic process	2	0.904977	0.040938	47.79487
13	negative regulation of axon regeneration	2	0.904977	0.040938	47.79487
14	activation of cysteine-type endopeptidase activity involved in apoptotic process	2	0.904977	0.040938	47.79487
15	response to bacterium	3	1.357466	0.041002	9.25062
16	steroid hormone mediated signaling pathway	4	1.809955	0.043213	5.09812
17	transcription, DNA-templated	14	6.334842	0.047646	1.791508
18	retina morphogenesis in camera-type eye	3	1.357466	0.048484	8.434389

GO analysis of 168 differentially expressed transcripts under germ-free conditions compared to monoassociated with *Chryseobacterium* (ZOR0023) (see supplementary table 4).

Rank	GO Term	Number of genes	Percentage of total	P-value	Fold enrichment
1	regulation of transcription from RNA polymerase II promoter	10	6.535948	1.44E-04	5.096377
2	regulation of transcription, DNA-templated	22	14.37908	4.65E-04	2.276641
3	regulation of cell growth	4	2.614379	0.002867	13.90396
4	negative regulation of cell proliferation	4	2.614379	0.004313	12.0501
5	apoptotic process	5	3.267974	0.012616	5.510717
6	regulation of MAP kinase activity	2	1.30719	0.02177	90.37576
7	activation of cysteine-type endopeptidase activity involved in apoptotic process	2	1.30719	0.028922	67.78182
8	intracellular signal transduction	7	4.575163	0.043367	2.711273
9	transcription, DNA-templated	11	7.189542	0.04656	1.996252
10	single organismal cell-cell adhesion	3	1.960784	0.049842	8.299814

GO analysis of 122 differentially expressed transcripts under germ-free conditions compared to monoassociated with *Exiguobacterium* (ZWU0009) (see supplementary table 5).

Rank	GO Term	Number of genes	Percentage of total	P-value	Fold enrichment
1	regulation of transcription from RNA polymerase II promoter	8	7.54717	2.44E-04	6.316637
2	cell-matrix adhesion	4	3.773585	0.001301	18.26332
3	response to bacterium	3	2.830189	0.009251	20.32531
4	regulation of cell growth	3	2.830189	0.014391	16.15601

**5**	epiboly involved in gastrulation with mouth forming second	3	2.830189	0.016571	15.00201
**6**	cell adhesion	6	5.660377	0.016733	3.962796
**7**	regulation of transcription, DNA-templated	13	12.26415	0.018069	2.084249
**8**	mitochondrial transport	2	1.886792	0.032407	60.00805

GO analysis of 84 differentially expressed transcripts under germ-free conditions compared conventionalized in the *myd88* deficient mutant background (see supplementary table 7).

Rank	GO Term	Number of genes	Percentage of total	P-value	Fold enrichment
**1**	lipid transport	6	8.571429	4.32E-06	24.11644205
**2**	oxidation-reduction process	12	17.14286	7.05E-06	5.481009556
**3**	reverse cholesterol transport	3	4.285714	6.43E-04	76.73413379
**4**	cholesterol efflux	3	4.285714	0.001387	52.75471698
**5**	transport	12	17.14286	0.00141	3.014555256
**6**	lipoprotein metabolic process	3	4.285714	0.002879	36.69893355
**7**	regulation of intestinal cholesterol absorption	2	2.857143	0.024161	80.38814016
**8**	positive regulation of fatty acid biosynthetic process	2	2.857143	0.024161	80.38814016
**9**	positive regulation of lipoprotein lipase activity	2	2.857143	0.024161	80.38814016
**10**	phosphatidylcholine metabolic process	2	2.857143	0.027565	70.33962264
**11**	lipid metabolic process	4	5.714286	0.028513	5.923336643
**12**	triglyceride homeostasis	2	2.857143	0.030958	62.52410901
**13**	folic acid-containing compound biosynthetic process	2	2.857143	0.030958	62.52410901
**14**	high-density lipoprotein particle assembly	2	2.857143	0.030958	62.52410901
**15**	very-low-density lipoprotein particle remodeling	2	2.857143	0.030958	62.52410901
**16**	positive regulation of cholesterol esterification	2	2.857143	0.030958	62.52410901
**17**	positive regulation of triglyceride catabolic process	2	2.857143	0.034339	56.27169811
**18**	phospholipid efflux	2	2.857143	0.037709	51.15608919
**19**	cholesterol homeostasis	2	2.857143	0.037709	51.15608919
**20**	neuron projection regeneration	2	2.857143	0.041067	46.89308176
**21**	triglyceride catabolic process	2	2.857143	0.041067	46.89308176
**22**	arachidonic acid metabolic process	2	2.857143	0.044414	43.28592163
**23**	cholesterol biosynthetic process	2	2.857143	0.047749	40.19407008

Supplementary Table 8

7 transcripts associated with either macrophages (M) or neutrophils (N) were regulated in response to conventionalization in WT (ABTL) larvae. 4 transcripts associated with macrophages or neutrophils were regulated in response to *Myd88* deficient mutants (mut).

Gene name	Expressed in Magrophages or Neutrophils	Regulated in response to conventionalization in WT (ABTL) or in Myd88 mutant (mut)
**mpeg1.2**	M/N	**ABTL/mut**
**cebpb**	M/N	**ABTL**
**myd88**	M/N	**ABTL**
**csrnp1a**	M/N	**ABTL**
**socs1a**	M/N	**ABTL**
**lta4h**	M/N	**mut**
**cd36**	M/N	**mut**
**irg1l**	M	**ABTL**
**slc7a7**	M	**mut**
**nr4a3**	N	**ABTL**

Supplementary references

1. Rhodes, J. *et al.* Interplay of pu.1 and Gata1 determines myelo-erythroid progenitor cell fate in zebrafish. *Dev. Cell* **8,** 97–108 (2005).
